# Dual inhibition of atypical PKC signaling and PI3K/Akt signaling dysregulates c-Myc to induce apoptosis in clear cell Renal Cell Carcinoma

**DOI:** 10.3389/fonc.2023.1213715

**Published:** 2024-01-15

**Authors:** Khandker Mohammad Khalid, Wishrawana S. Ratnayake, Christopher A. Apostolatos, Mildred Acevedo-Duncan

**Affiliations:** Department of Chemistry, University of South Florida, Tampa, FL, United States

**Keywords:** renal cell carcinoma, ICA-1, BYL719, PKC-ι, PKC-ζ, apoptosis

## Abstract

**Background:**

Renal Cell Carcinoma (RCC) is the most common type of kidney cancer (85%). 75% of the RCC cases involve conventional clear cell RCC (ccRCC). Approximately, 39% of late-stage patients (stage IV) are treated with chemotherapeutic agents. Phosphatidylinositol-3-kinase (PI3K) and Mitogen-Activated Protein Kinase Kinase (MEK)/extracellular signal-regulated kinase (ERK1/2) pathways are frequently activated in RCC. In addition, atypical PKCs (PKC-ί and PKC ζ) are overexpressed in most cancer cells, and they play a central role in tumor progression and the metastasis of different types of cancers. Our goal is to establish the role of aPKCs in the regulation of multiple key activated pathways in ccRCC. In this study, we also established a novel therapeutic regimen for dual inhibition of key activated pathways.

**Method:**

In this study, 786-0 and Caki-1 cells were studied and subjected to cell viability assay, western blot analysis, scratch & wound healing assay, transwell invasion assay, immunofluorescence, immunoprecipitation, flow cytometry, and quantitative real-time polymerase chain reaction. We used combination of PI3K inhibitor- Alpelisib (BYL719) and ICA-1 (a PKC-ι-specific 5-amino-1-2,3-dihydroxy-4-(methylcyclopentyl)-1H-imidazole-4-carboxamide). In addition to drug treatment, small interfering RNA (siRNA) technology was used to further confirm the experimental outcome of the drug treatment.

**Results:**

Our results suggest that treatment of ccRCC cells with a combination of ICA-1 (aPKC inhibitor) and BYL719 (PI3K inhibitor) downregulates PKC-ί and causes downstream inhibition of c-Myc. Inhibition of the PKCί also reduces activation of MEK/ERK1/2. It is observed that treatment with ICA-1 disrupts the level of the aPKC-Akt1 association. ICA-1 treatment also shows a reduced level of association between aPKC and c-Myc. The inhibition of aPKCs and downstream effector proteins by combination therapy is more pronounced compared to a single therapy. These effects contribute to reduced cell growth, and eventually, the induction of apoptosis. The decreased level of N-cadherin, p-vimentin, and vimentin and the increased level of E-cadherin confirm reduced malignancy.

**Conclusion:**

Therefore, implementing a combination of Alpelisib and a PKC-ι inhibitor is an effective approach to reducing cell proliferation, and invasion that eventually induces apoptosis and may be considered as a potential therapeutic option in ccRCC.

## Introduction

Kidney and Renal Pelvic cancer is the sixth most common cancer among males and the ninth most common cancer among females. Renal Cell Carcinoma (RCC) is the prevalent type of kidney cancer (85%); Clear cell RCC (ccRCC) is the predominant subtype (75%) of all the cases (1). Although partial or radical nephrectomy is the primary choice of treatment for early stages (stage I and II), recurrent RCC who have already undergone nephrectomy or are at the later stages of RCC (stage II and IV), chemotherapy is one of the most viable options to eradicate RCC or aiding a progression-free survival (PFS) (1, 2).

Choices of treatments recommended for clear cell carcinoma and non-clear cell carcinoma include partial nephrectomy (Stage I), radical nephrectomy (Stages II and III), and chemotherapy (stage IV). As RCC is recurrent, cases have been reported that reflect cutaneous metastasis several years after radical nephrectomy (2). As such, chemotherapy is the next choice of treatment for patients who have already undergone nephrectomy. This type of cancer stems from the cell lining of the small tubules that usually have either a genetic mutation of a hereditary VHL (Von Hippel-Lindau) or a sporadic mutation in other genes. For two decades, various strategies have been devised to address the VHL insufficiency due to genetic mutation that results in constitutive stabilization of hypoxia-inducible factor (HIF)-1α and HIF-2α (3).

Frequently, amongst other relevant pathways, these genetic mutations are involved with the downstream activation of the Phosphatidylinositol 3 kinase (PI3K)/Protein Kinase B (Akt)/mammalian target of Rapamycin (mTOR) pathways. The PI3K/Akt/mTOR pathway is modestly mutated (in 27.7% of the cases) and highly activated in ccRCC. In a complex signaling network, the VHL/HIF and PI3K/Akt pathways interact often, and they are associated with ccRCC (4).

MYC activation plays a central role in and its overexpression drives renal cell carcinoma (5, 6). When combined with VHL and Cdkn2a deletion, the overexpression of c-Myc, is reported to produce kidney tumors (7). c-Myc is reported to be the center of multiple pathways, and it plays a pivotal role in cell proliferation, survival, invasion, and the apoptosis in ccRCC. MYC overexpression occurs rarely due to a direct mutation, rather, it is typically the result of upstream oncogenic signaling (8, 9). Despite the pivotal role that c-Myc plays in ccRCC, no primary sequence that identifies the active site could be identified. This limits the development of the small molecule antagonists of c-Myc (10). Alternatively, the interruption of direct protein-protein interaction involving c-Myc and its co-activators, such as ERK1/2 and the components of PI3K/Akt, can abrogate the transcriptional activity of c-Myc.

Multiple attempts have been made to pharmacologically modulate the PI3K/Akt pathway to tackle ccRCC, and these have been published in previous studies (11, 12). In addition, mTOR signaling proteins have been associated with the major target signaling pathways of this type (13). mTOR inhibition using rapamycin is reported to induce upstream receptor tyrosine kinase signaling, and it also produces rapamycin induced Akt activation (14). By negatively regulating GSK-3β, the PI3K/Akt pathway imparts protein stability to c-Myc.

In addition, the ERK1/2-mediated phosphorylation of Serine 62 in c-Myc enhances the protein stability of c-Myc (15). Moreover, PKC-ι is also reported to control the activation of the MEK/ERK axis (16).

PKC-ι was first proposed as a novel therapeutic target to treat lung cancer (17). There are fifteen types of PKCs found in humans, placed in three categories (classical, novel, and atypical PKCs). Of these, the atypical Protein Kinase Cs (aPKCs) have various isoforms (18, 19). Enzymes belonging to the atypical protein kinase C (PKC) family represent one of the major mediators of signal transduction in melanocytes, glioma, as well as ovarian, lung, and colorectal carcinoma. Atypical protein kinase Cs (aPKC) are involved in the cell cycle progression, tumorigenesis, cell survival and migration in many cancers (20–24). Thus, in this study, we investigated the central role that PKC-ι played in Akt1 and ERK1/2 activation. We used ICA-1 (1H-imidazole-4-carboxamide, 5-amino-1-[2,3-dihydroxy-4-[(phosphonooxy) methyl] cyclopentyl]-, [1R-(1a, 2b, 3b, 4a)]), a selective PKC-ι inhibitor. ICA-1 has been poven to significantly modulate many pivotal oncogenic pathways by selcetively inhibiting PKC-ι (22, 23, 25, 26).

BYL is a selective PI3Kα inhibitor developed by Novartis, and it is currently approved for HR+/HER2- advanced breast cancer. BYL underwent a phase Ib clinical trial as a combination drug with Everolimus for various cancer types, including metastatic and/or recurrent solid tumors such as renal cell carcinoma (RCC) (27). In addition, BYL719 has been reported to be effective against malignancies that have unmutated *PI3KCA* genes (28). Using a single PI3K inhibitor, surprisingly, may also result in enhanced ERK1/2 phosphorylation, which is crucial for c-Myc stability; this renders the treatment ineffective (29). Therefore, we proposed using the combination of the novel selective inhibitor of PKC-ι (ICA-1) and the selective PI3Kα inhibitor Alpelisib (BYL719).

We hypothesized that PKC-ι plays a central role in multiple converging and bifurcating pathways, and that it controls the stability of c-Myc overexpression, which is a master regulator of oncogenic signaling. We investigated the dual inhibition of PI3K and PKC-ι with BYL and ICA-1 to determine its effectiveness against cell viability, apoptosis, invasion, and the migration of RCC by indirectly targeting c-Myc.

## Results

### BYL719 combined with ICA-1 exhibit increased cytotoxicity in both Caki-1 and 786-0 cell lines

**Figure 1:**
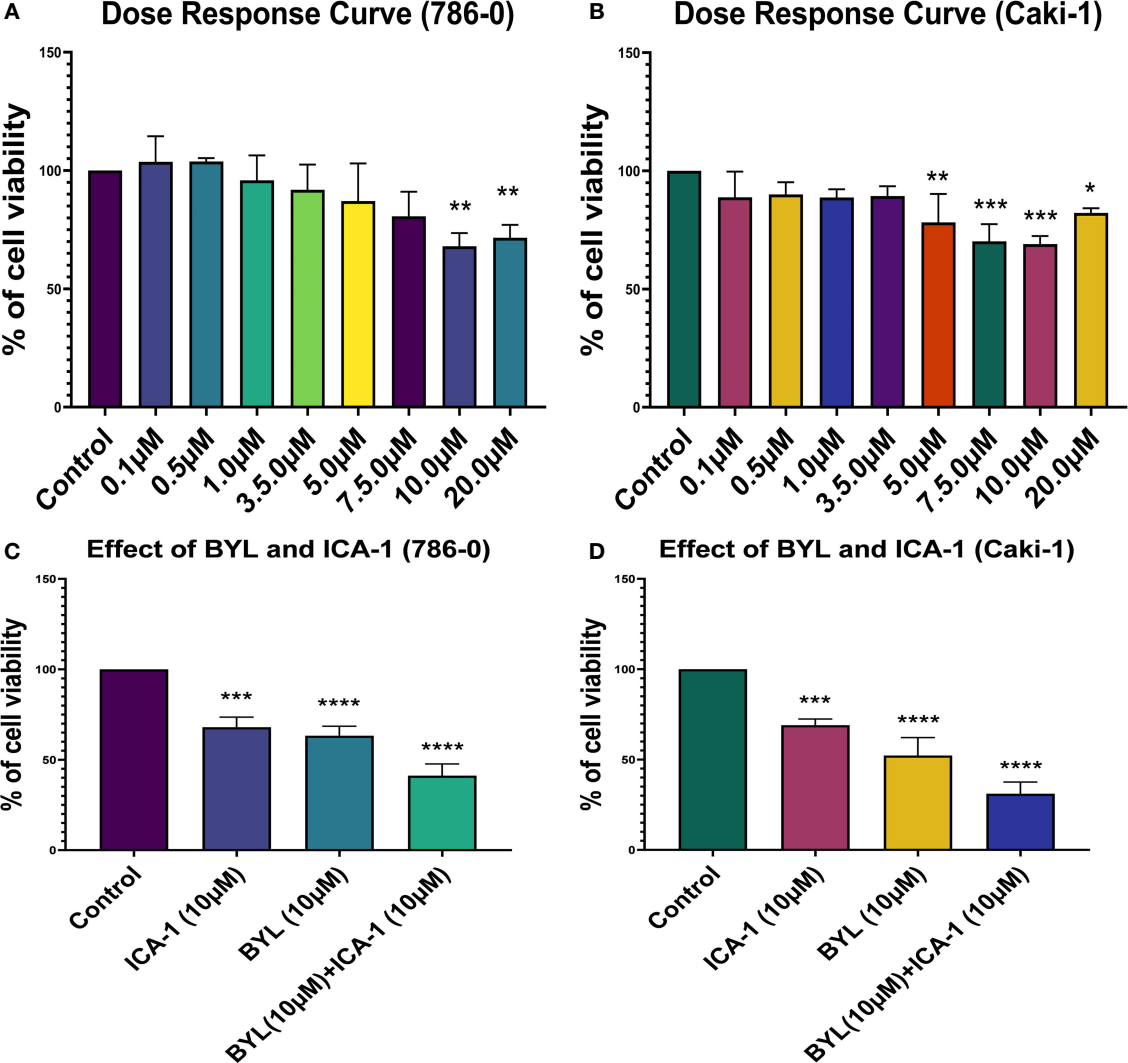
Cell Viability assays in Caki-1 cell line using various doses of ICA-1, BYL719 and combination therapy. **(A)** 786-0 cells were treated with increasing doses of ICA-1 for three consecutive days. **(B)** 786-0 cells were treated with increasing doses of ICA-1 for three consecutive days. **(C)** 786-0 cells were treated with BYL719 (10.0µM) with a subsequent combination of ICA-1 at a dose of 10µM. **(D)** Caki-1 cells were treated with BYL719 (10.0µM) with a subsequent combination of ICA-1 at a dose of 10µM. Viable cells were measured after three days of treatment after incubating with WST-1 for 3h and subsequent determination of absorbance at 480nm. The absorbance of the control group was taken as 100% viable. The data represent *N*=3 independent experiments, mean ± Standard Error of Mean (SEM). (* indicates *P*≤0.05, ** indicates *P*≤0.01, *** indicate *P*≤0.001, **** indicates *P*≤0.0001).

Three days of treatment of 786-0 and Caki-1 cells with ICA-1 inhibits cell viability in a dose-dependent manner. From the dose-response curve, a 30% reduction of cell viability was observed at a concentration of 10.0 µM in 786-0 (*P*<0.01) and Caki-1 (*P*<0.01) cells. We chose these doses to treat the cells as part of a combination therapy with BYL719 (10.0 µM). Based on the results observed, the cytotoxicity of cells subjected to combination therapy was significantly (*P*<0.001) higher in both cells. For ICA-1 combined with BYL719, the cytotoxicity was increased almost 20% compared to the BYL719 monotherapy. The Coefficient of Drug Interaction (CDI) value for the combination therapy was <1, which shows the drug combination has a synergistic effect on 786-0 (0.95) and Caki-1 (0.86) cell viability (**Figure 1**).

### Annexin-V-FITC and PI Analysis revealed increased apoptosis in the BYL719 and ICA-1 combination therapy

An Annexin-V assay was performed in both the Caki-1 and 786-0 cell lines to determine whether the drug treatment induces apoptosis in the cultured cells. For the Caki-1 cell line, the control group had 90% live cells, with 0.7% cells undergoing early apoptosis and 4.3% undergoing late apoptosis. Treatment with only ICA-1 (10µM) induced 11% early apoptosis. In turn, ICA-1 single therapy did not bring any notable late apoptosis (1.03%) compared to the control. BYL719 (10µM) monotherapy in Caki-1 resulted in 7.23% (*P*<0.05) early apoptosis and 8.23% (*P*<0.01) late apoptosis, respectively.

**Figure 2:**
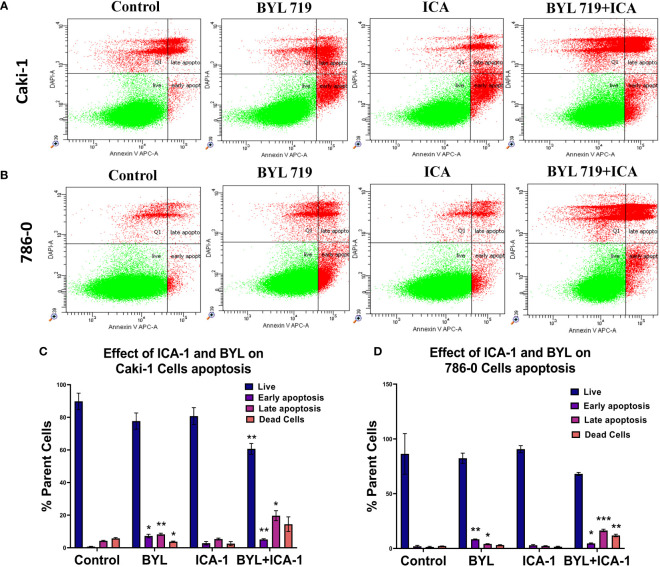
Measurement of apoptosis via Annexin V-FITC/PI staining. Representative dot plot expresses the total number of cells of different treatment groups represented as a percentage of healthy cells, early apoptotic cells, late apoptotic cells, and dead cells. **(A)** Caki-1 left untreated or treated with ICA (10 µM), BYL719 (10µM), BYL719 (10µM) + ICA-1 (10 µM) for three consecutive days **(B)** 786-0 cells left untreated or treated with ICA-1 (10µM), BYL719 (10µM), BYL719 (10µM) + ICA-1 (10 µM) for three consecutive days. **(C**, **D)** are graphical representations of apoptosis on Caki-1 and 786-0. The data represent *N*=3 independent experiments, mean ± Standard Error of Mean (SEM). (* indicates *P*≤0.05, ** indicates *P*≤0.01, *** indicate *P*≤0.001).

Subsequently, combination treatment with BYL719 (10µM) and ICA-1 (10µM) caused a greater number of cells (19.7%) to undergo late apoptosis (*P*<0.05) (**Figure 2**).

The assay for the 786-0 cell line revealed that utilizing BYL719 (10µM) and ICA-1 (10µM) for combination treatment increased the number of cells undergoing late apoptosis (**Figure 2**). The control group had 86% live cells along with only 2% cells with early apoptosis and 1.37% cells with late apoptosis. Treatment with ICA-1 did not notably increase the early apoptotic cells (0.89%). Treatment with BYL719 (10 µM) resulted in a decreased number of live cells (82.4%) compared to the control group, and this resulted in an increased number of cells undergoing early (8.37%) (*P*<0.01) and late apoptosis (4.07%) (*P*<0.05). Combination treatment with BYL719 (10µM) and ICA-1 (10µM) decreased the number of cells undergoing early apoptosis (4.57%) (*P*<0.05), and it significantly increased late apoptosis (16.4%) (*P*<0.001).

### Dual inhibition of PKC-ι and PI3K pathways signaling resulted in increased apoptosis

**Figure 3:**
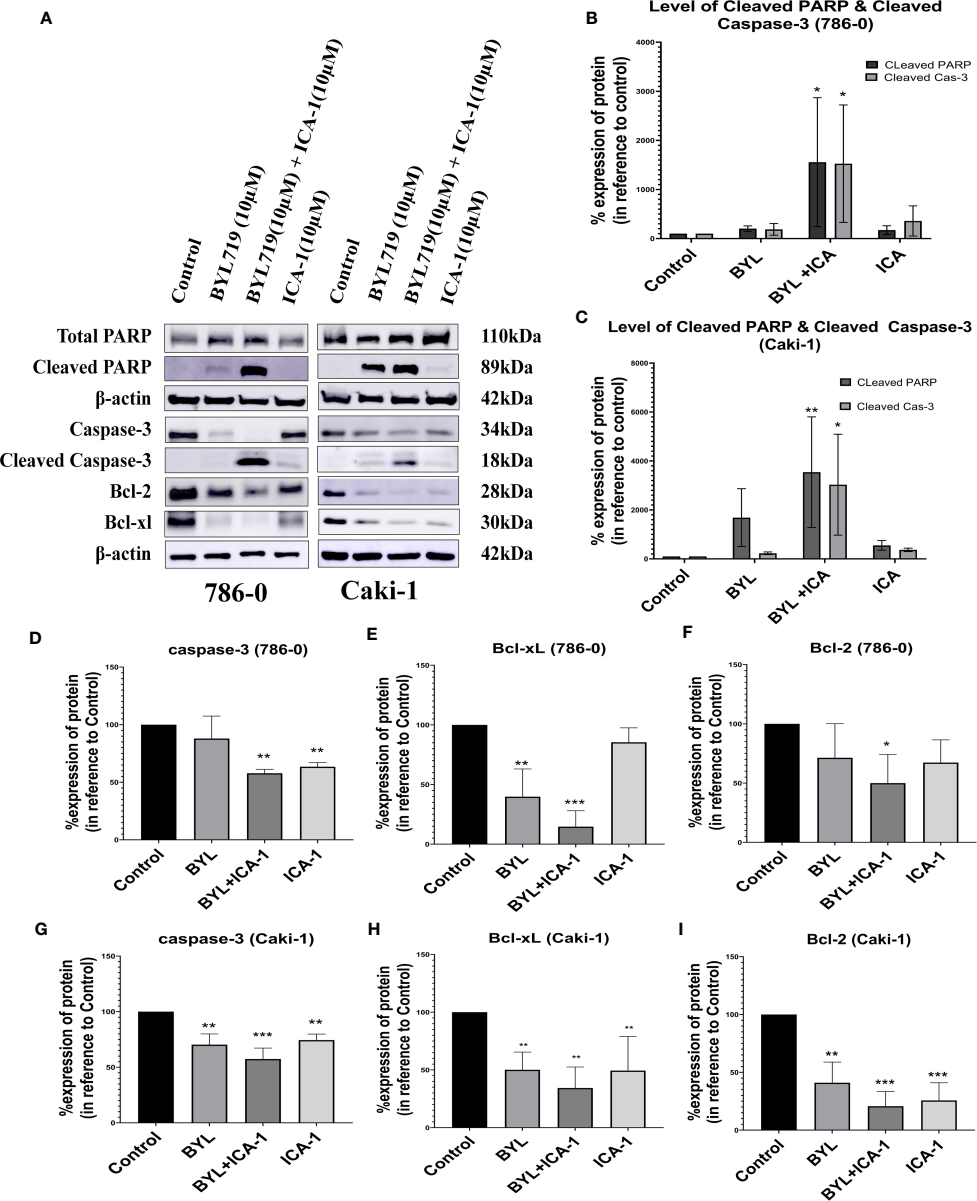
Measurement of Apoptosis via western blot analysis in Caki-1 and 786-0 cells. **(A)** Western blot analysis and the effect of ICA-1, BYL and BYL combined with either ICA-1 following three days of treatment on **(B)** cleaved Caspas-3, **(C)** cleaved PARP, Caspase-3, PARP, Bcl-xL and Bcl-2 protein in 786-0 and Caki-1 cells **(D–I)**. The data represent *N*=3 independent experiments, mean ± SEM. (* indicates *P ≤* 0.05, ** indicates *P ≤* 0.01, *** indicate *P ≤* 0.001).

The combination of BYL719 and ICA-1 promoted apoptosis in both cell lines, as demonstrated by the changes in the apoptotic protein markers. Our findings showed that combining BYL with ICA-1 resulted in a considerable increase in the level of cleaved Caspase-3 and the cleaved PARP expression in both the RCC cell lines. (**Figure 3**). The treatment group of BYL combined with ICA-1 exhibited greater caspase cleavage and PARP cleavage, indicating that this treatment group resulted in the highest level of apoptosis. The treatment of ICA-1 and BYL also resulted in a decreased level of Caspase-3 proteins in both the 786-0 and Caki-1 cells with statistical significance (*P ≤* 0.05). The combination of BYL with ICA-1 significantly decreased the levels of the pro-survival B cell lymphoma-2 (Bcl-2) proteins and the B-cell lymphoma-extra large (Bcl-xL) proteins in both the cell lines (*P ≤* 0.05; **Figure 3**). This research found that inhibiting PKC-ι, in conjunction with PI3K, promoted apoptosis and that PKC-ι can be considered as a therapeutic target in the treatment of ccRCC.

These results clearly indicated that the combination of BYL with ICA-1 significantly induced apoptosis compared with Alpelisib (BYL719) monotherapy in both cell lines (**Figure 3**).

### Alpelisib (BYL) combined with ICA-1 significantly reduces p-PKC-ι and PKC-ι

**Figure 4:**
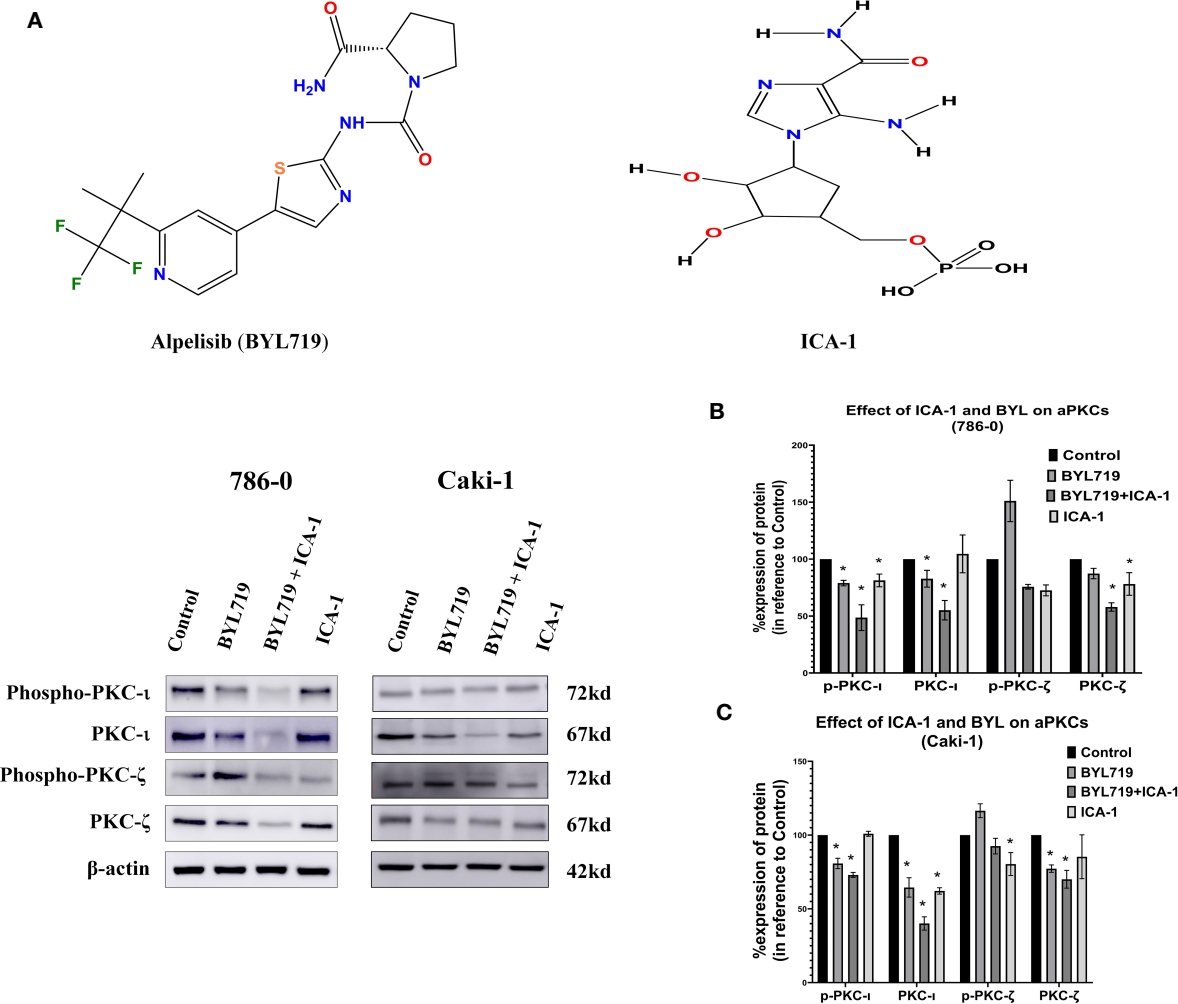
Effect of a-PKC inhibitors, BYL719 and combination therapy on a-PKCs in 786-0 and Caki-1 cells. **(A)** Western blot analysis of a-PKCs in 786-0 and Caki-1. The bar graphs **(B, C)** indicate the densitometry of the total PKCs and p-PKCs as a percentage of control. The data represent *N*=3 independent experiments, mean ±SEM. (* indicates *P*≤0.05).

The results observed from the western blot analysis show that the in Caki-1 cells ICA-1 treatment have a significant reduction (*P*<0.05) in the phosphorylation of PKC-ι(T 555) and p-PKC−ζ(T-410). In Caki-1 cells, ICA-1 reduces both p-PKC−ζ(T-410) and PKC-ζ. However, the results of the BYL and ICA-1 combination therapy show a significant reduction (*P*<0.05) in p-PKC-ι(T 555), PKC−ι and PKC-ζ levels. The p-PKC−ζ(T-410) level for combination therapy is not significantly reduced compared to the ICA-1, but it was lower than it was for the BYL monotherapy. In 786-0 cells, ICA-1 significantly reduces (*P*<0.05) the p-PKC-ι(T 555), PKC-ι and PKC-ζ levels. The combination therapy of BYL with ICA-1 in 786-0 reduced the PKC-ι phosphorylation to 20%. In turn, the PKC-ι level was reduced to 37% compared to the control group. The PKC-ζ level was reduced to 44% (**Figure 4**).

### Combination of BYL and ICA-1 reduces c-Myc level via deactivation of Ak1 and ERK1/2

**Figure 5:**
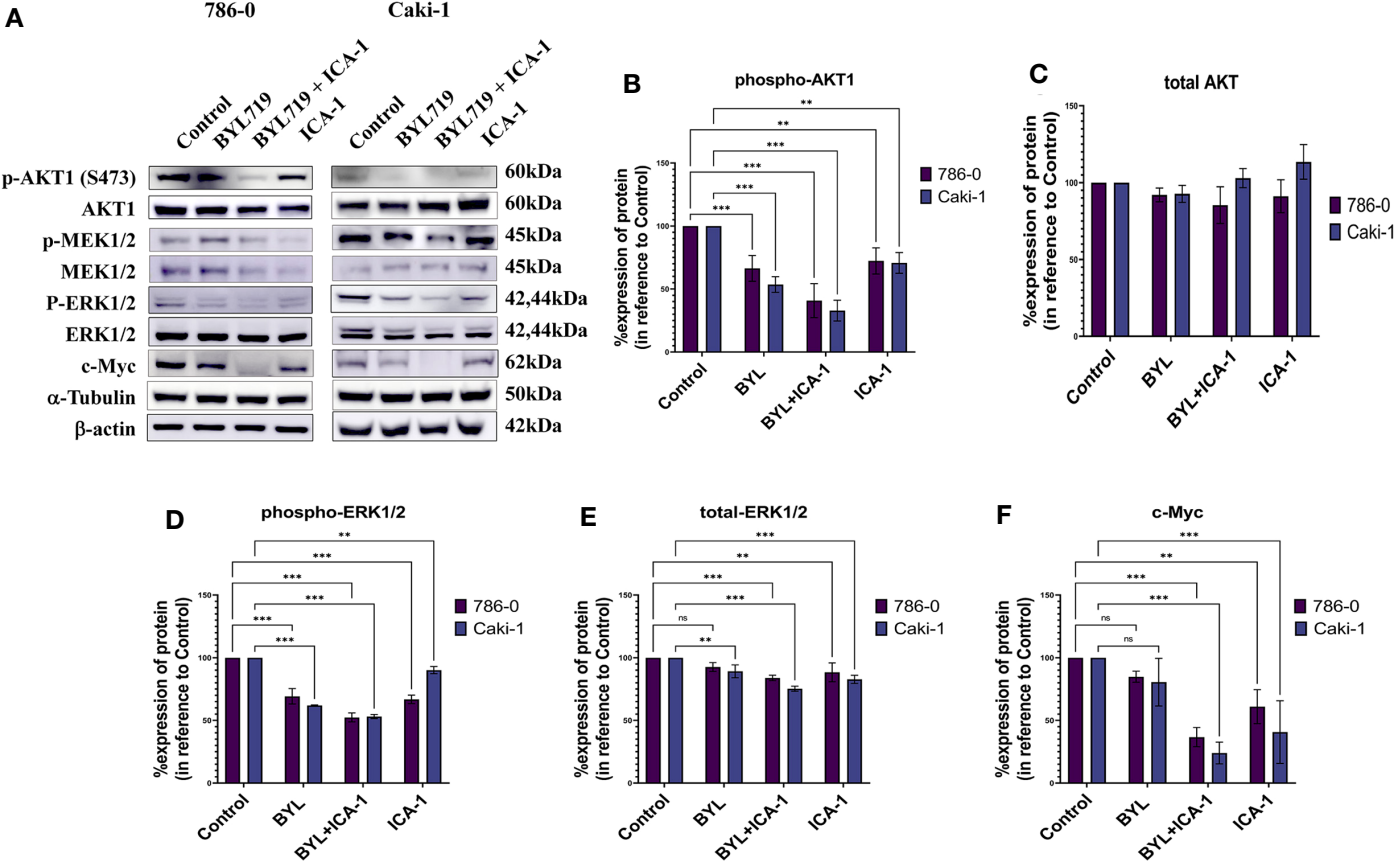
Effect of aPKC inhibitors ICA-1, BYL719 and combination therapy on c-Myc regulation in Caki-1 and 786-0 Cells **(A)** Western blot analysis and the effect of ICA-1,BYL719 and BYL719 combined with ICA-1 following three days of treatment on p-Akt1, Akt1, p-ERK1/2, ERK1/2, and c-Myc. **(B–F)** show the bar diagrams of the densitometry of p-Akt1, Akt1, p-ERK1/2, ERK1/2, and c-Myc in 786-0 and Caki-1. In both case the data represent *N*=3 independent experiments, mean ±SEM. (ns indicates Not Significant, ** indicates *P*≤0.01, *** indicate *P*≤0.001).

After treating the cells for three days, Western Blotting was performed to evaluate the protein levels of p-Akt (S473), Akt1, p-MEK1/2, MEK1/2, p-ERK1/2, ERK1/2, and c-Myc. The results indicated that the combination treatment of BYL and ICA-1, which caused a significant decrease in PKC-ι activation, resulted in an almost 60% decline in p-Akt1(Ser473) levels in both cell lines (**Figures 5A, B**). However, drug treatment with single or combination treatment did not cause any significant change in the total-Akt level (**Figures 5A, C**).

Phospho-ERK1/2 expression is also reduced significantly to almost 53% (*P<0.001*) in both cell lines following ICA-1 and BYL treatment. The individual ICA-1 treatment resulted in a 67% decrease of ERK1/2 activation in the 786-0 cell line; in contrast, in Caki-1 cells, the reduction is 90% (*P*<0.01) compared to the control group. BYL monotherapy produced a 62% (*P*<0.001) and a 69% (*P*<0.001) decrease in ERK1/2 activation in the 786-0 and Caki-1 cells, respectively (**Figures 5A, D**).

Monotherapy with BYL did not produce a significant reduction in the total ERK1/2 in 786-0 cells compared to the control group. In Caki-1 cells, a total decrease of ERK1/2 was found to be less but significant (*P*<0.01) when compared to the control group. In both cell lines, ICA-1 therapy resulted in a 88% (*P*<0.001) and a 83% (*P*<0.001) decrease in 786-0 and Caki-1 cells, respectively (**Figures 5A, E**).

Similarly, the combination of ICA-1 and BYL brings the c-Myc level down to less than 37% and 24% in the Caki-1 and 786-0 cell lines, respectively (*P<0.001*). However, single BYL therapy did not cause a significant reduction in the c-Myc level, while we observed a decrease in the c-Myc level in 786-0 and Caki-1 cells (61% and 41%, respectively) (*P*<0.01) as a result of ICA-1 monotherapy (**Figures 5A, F**). These data suggests that the combination of BYL and ICA-1 brings a notable alteration in the level of c-Myc by deactiovationg its upstream effector Akt1 and ERK1/2.

### siRNA knockdown of PKC-ι results in deactivation of Akt1, and ERK1/2 and decreased c-Myc level

**Figure 6:**
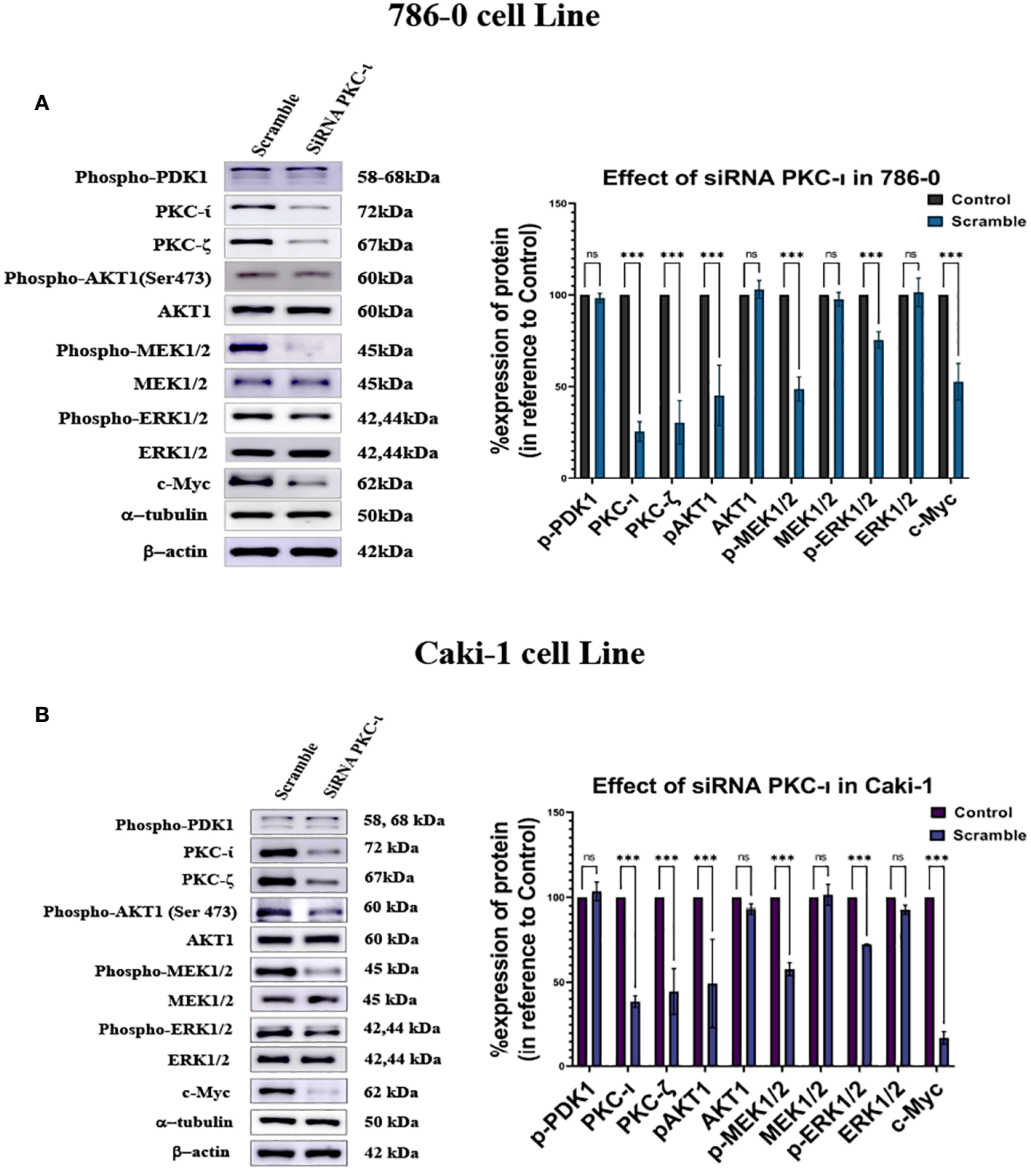
Effect of siRNA-PKC- in Caki-1 and 786-0 Cells on c-Myc regulation **(A)** Western blot analysis was done after treating the cells with 25nm siRNA PKC-ι and the cell lysates were probed for PKC-ζ, p-Akt1, Akt,p-MEK1/2, MEK1/2, p-ERK1/2, ERK1/2, and c-Myc in 786-0 cells **(B)** Western blot analysis was done after treating the cells with 25nm siRNA PKC-ι for and the cell lysates were probed for PKC-ζ, p-Akt1, Akt1,p-MEK1/2, MEK1/2, p-ERK1/2, ERK1/2, and c-Myc in Caki-1 cells. In both cases, the data represent *N*=3 independent experiments, mean ±SEM. (ns indicates Not Significant, * indicates *P*≤0.05, ** indicates *P*≤0.01, *** indicate *P*≤0.001).

It was observed that the knockdown of PKC-ι resulted in a decrease in the c-Myc expression in both the Caki-1 (17.3%; *P*<0.001) and 786-0 (53%; *P*<0.001) cell lines (**Figures 6A, B**). This may be due to a concerted effect of reduced activation of Akt1 (Ser473) and ERK1/2 and a result of PKC-ι knockdown. The results show that Caki-1 cells have about a 51% (*P*<0.001) knockdown of p-Akt1 (Ser473) and a 27.8% (*P*<0.001) decreased activation of p-ERK1/2 (**Figure 6B**). In the 786-0 cells, the PKC-ι knockdown resulted in a 54% (*P*<0.001) decrease activation of Akt1 and about a 24.5% (*P*<0.001) decrease in p-ERK1/2. (**Figure 6A**). Similar to drug treatment, siRNA treatment, in both the Caki-1 and 786-0 cells caused about a 75% (*P*<0.001) and a 61% (*P*<0.001) knockdown of PKC-ι, respectively, triggering the downstream deactivation of Akt1 and ERK1/2. Both the Caki-1 and 786-0 cells show a significant reduction of c-Myc (*P*<0.001). In the Caki-1 cells, the decrease is more pronounced (85%); in the 786-0 cells, there is an almost 37% reduction of the c-Myc level. The results also show that PKC-ι knockdown with siRNA reduces the PKC-ζ expression in both Caki-1 (55% reduction) and 786-0 (70% reduction) (*P*<0.001) (**Figure 6**).

### Immunoprecipitation of PKC-ι showed association with Akt1 and c-Myc

**Figure 7:**
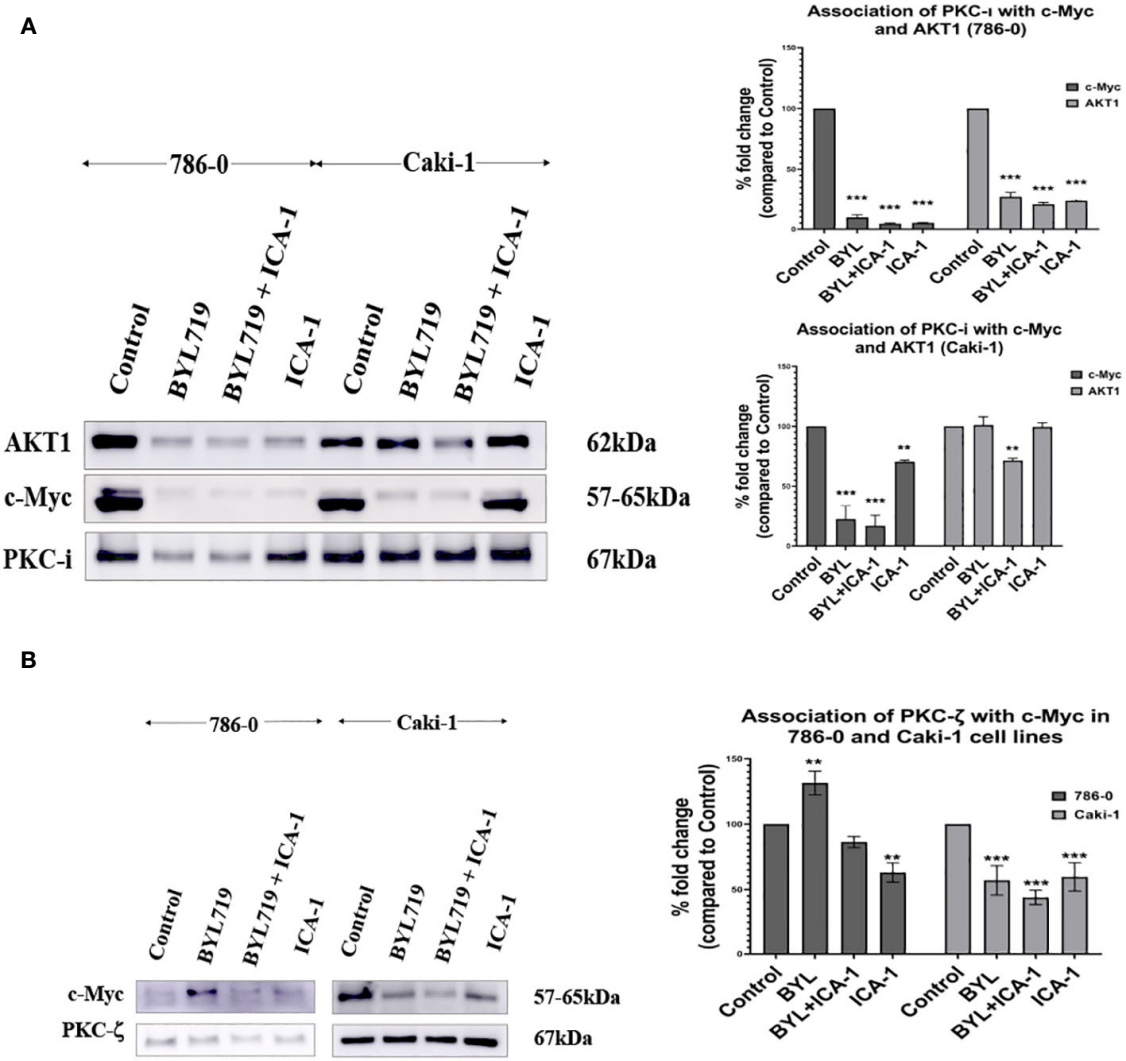
**(A)** PKC-ι is associated with Akt1 and c-Myc in both Caki-1 and 786-0 cell lines BYL719 combined with ICA-1 (10µM) decrease the association between PKC-ι and Akt1. Caki-1 and 786-0 Cells were treated with drugs and lysate was collected, PKC-ι was pulled down from 1000µg protein lysates. The pulled-down protein was subjected to SDS page and immunoblotted for Akt1, c-Myc and PKC-ι. BYL719 combined with ICA-1 reduced the level of association between PKC-ι and Akt1. PKC-ι and c-Myc association is also markedly reduced by the combination therapy. The bar diagram on the right shows the comparison of the associated Akt1 and c-Myc proteins with PKC-ι pulldown. The data represent *N*=3 independent experiments, mean ±SEM. (* indicates *P*≤0.05). **(B)**: PKC-ζ is associated with c-Myc in both 786-0 and Caki-1 cells. The data represent *N*=3 independent experiments, mean ±SEM. (** indicates *P*≤0.01, *** indicate *P*≤0.001).

**Figure 8:**
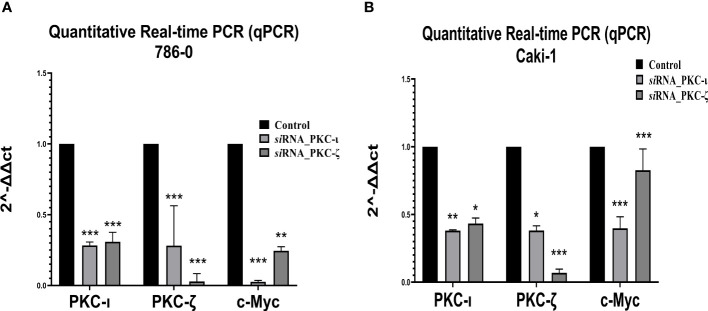
mRNA levels of PKC-ι, PKC-ζ, and c-Myc for the siRNA (PKC-ι and PKC-ζ) treated **(A)** 786-0 and **(B)** Caki-1 cells against respective control samples based on quantitative real-time PCR (qPCR). The ΔΔCT values were plotted with respect to the mRNA levels of control samples of each cell line. *N* = 3 experiments were carried out. All values are reported as the means ± SD. Statistical significance is indicated by an asterisk (* indicates *P*≤0.05, ** indicates *P*≤0.01, *** indicate *P*≤0.001).

We performed an immunoprecipitation (IP) study of PKC-ι to evaluate the interaction of c-Myc because the upstream and downstream protein molecules must associate with each other to establish a signaling cascade. An agarose conjugated PKC−ι antibody was used to pull down PKC-ι from the cell lysates. An immunoblot analysis showed that PKC-ι was associated with Akt1 and c-Myc and that reduced level of associated c-Myc was observed with the combination treatment of BYL719 and ICA-1 (*P*<0.001) compared to the control group (by 83.1%) and by 25% compared to the single BYL therapy in the Caki-1 cells. In 786-0 cells, the PKC-ι associated c-Myc level is also reduced drastically (*P*<0.001; **Figure 7A**) by the combination treatment. A similar significance was observed in the reduction level of the associated Akt1 with IP PKC-ι in 786-0 and Caki-1 cells (79% and 29%, respectively) (*P*<0.01; **Figure 7A**). These data suggests that the combination of BYL and ICA-1 might bring a notable alteration in the level of associated c-Myc by deactivating its upstream effector Akt1 and ERK1/2. Transcriptional regulation of c-Myc via PKC-ι, which is evident by the data from qPCR (**Figure 8**), also plays role in a pronounced reduction of the total c-Myc level which finally results in decreased the level of c-Myc associated with PKC-ι. In both cells lines, PKC-ζ is also capable of associating with c-Myc. An immunoblot analysis showed that PKC-ζ was associated with c-Myc and that the combination treatment of BYL719 and ICA reduces the association with both PKC-ζ and c-Myc. The level of c-Myc associated with PKC-ζ was markedly decreased by combination therapy compared to the control group and monotherapy in the Caki-1 cells. (*P*<0.001; **Figure 7B**). Establishing the nature of interaction between aPKCs and c-Myc and the role of c-Myc as a part of the bigger complex merits further research.

### Simultaneous inhibition of PKC-ι and PI3K leads to decreased migration and invasion in RCC

**Figure 9:**
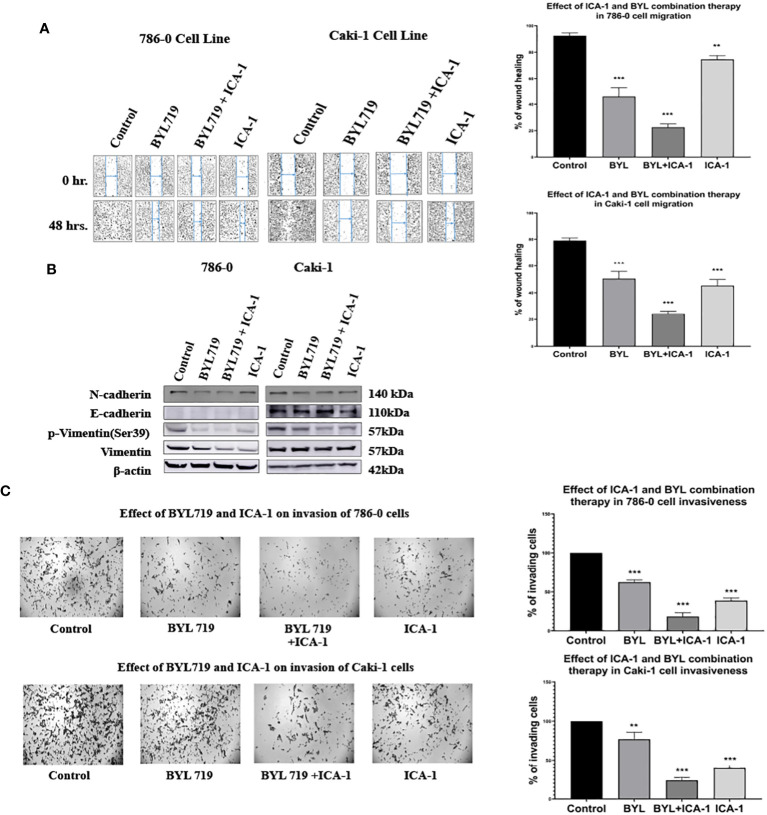
Effect of a-PKC inhibitors ICA-1, BYL and combination therapy on wound closure and cell invasiveness in 786-0 and Caki-1 Cells **(A)** Wound healing assay in 786-0 and Caki-1 cells after 48 h of treatment. The bar diagram shows the percentage of wound closure after 48 hours **(B)** Western blot analysis and the effect of ICA-1, BYL and BYL combination therapy on E-cadherin, N-Cadherin, p-vimentin, and vimentin. **(C)** Microscopic image of colony forming fields in the downward chamber in 786-0 and Caki-1 cells after 48 h of drug treatment. The bar diagram shows the percentage of invading cells in reference to control. In both cases, the data represent *N*=3 independent experiments, mean ± SEM. (** indicates *P*≤0.01, *** indicate *P*≤0.001).

To investigate the effects of the drugs and the combination treatment on cell migration, scratch assays were performed in both the Caki-1 and 786-0 cells. In the Caki-1 cells, after 48 hours of treatment, the wound healing was 79.1% in the control group. The BYL and ICA-1 combination treatment group showed an average wound healing rate of 24.5% (*P*<0.001). ICA-1 alone demonstrated wound closure of 45.3% (*P*<0.001). In the 786-0 cell line, the ICA-1 treated group demonstrated 74.6% wound healing (*P*<0.01) after 24 hours of drug treatment, compared to 92.5% in the control group. In turn, only 22.7% of wound healing was observed in 24 hours with BYL and ICA-1 combo treatment (*P*<0.001; **Figure 9A**).

A western blot analysis revealed that the combination drug treatment induced the downregulation of phospho-vimentin and vimentin, which coincided with the elevated expression of E-cadherin in the Caki-1 cell line. The reduced expression of N-cadherin was also observed with declines in the activated vimentin level in both the 786-0 and Caki-1 cells. These findings demonstrate that combination treatment with BYL and the PKC-ι inhibitor ICA-1 abrogates cell migration and invasion in ccRCC (**Figure 9B**).

Caki-1 cells displayed a similar reduction of invasive behavior in a Boyden chamber assay. A semiquantitative analysis using Image J revealed that in the Caki-1 cells, there was approximately a 60.4% decline (*P*<0.001) in invasion using ICA-1, while BYL on its own caused a 33% (*P*<0.01) decrease in invasion. The combination of BYL and ICA-1 showed a maximum decline in invasion with only a 76.2% reduction (*P*<0.001) in the number of invasive cells. These results reveal that ICA-1 reduces invasion by 60% (*P*<0.001) in the 786-0 cell line (**Figure 9C**).

In the BYL-only treatment group, invasion was reduced by 37.8% (*P*<0.001); in contrast, the combination of BYL and ICA-1 reduced invasion by nearly 81% (*P*<0.001) (**Figure 9C**).

### Quantitative real-time PCR and immunofluorescence microscopy confirms PKC-ι controls c-Myc stability

As **Figure 8** demonstrates, the qPCR data revealed that both the PKC-ι and PKC-ζ mRNA levels significantly decreased upon having been treated with siRNA of PKC-ι and PKC-ζ in both the tested cell lines (786-0 and Caki-1). In 786-0 cells, the mRNA of PKC-ι was reduced by 71.76% (P ≤ 0.001) and that of PKC-ζ by 71.83% (P ≤ 0.001) for the siRNA of PKC-ι. Similarly, the mRNA of PKC-ι was reduced by 69.16% (P ≤ 0.001) and by 97.09% of PKC-ζ (P ≤ 0.001) for the siRNA of PKC-ζ in 786-0 cell lines. A similar trend was observed in the Caki-1 cell line for both the siRNA treatments for PKC-ι and PKC-ζ. Since the specificities of siRNA for the particular targets were confirmed in the preliminary stages, the results indicated that the diminution of either PKC-ι, PKC-ζ in the 786-0 and Caki-1 cells disrupts the transcription of the other atypical PKC isoform. Interestingly, the m-RNA levels of c-Myc were significantly reduced in both cell lines upon the diminution of PKC-ι and PKC-ζ, as shown in **Figure 8**.

**Figure 10:**
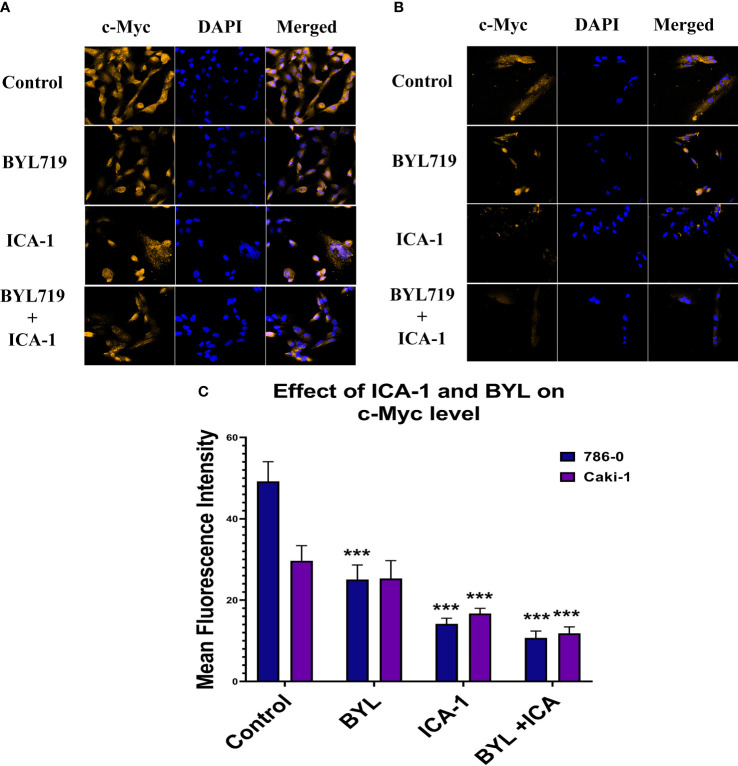
Effect of atypical PKC inhibitor (ICA-1), BYL, and combination therapy on c-Myc. 786-0 **(A)** and Caki-1 **(B)** cells were incubated for 72 h in the presence of atypical PKC inhibitors, BYL, and combination therapy. **(C)** Bar graph represents the relative abundance of c-Myc in the Control and treated cells. Cells were then fixed, and c-Myc was stained. Nuclei were counterstained with DAPI. Red represents c-Myc staining, and blue represents cell nuclei. Magnification is 4×10X. The bar diagram reflects the mean fluorescence intensity of the cells calculated by Image J software. (N=3) (*** indicate *P*≤0.001).

**Figure 10** represents the comparison of Immunofluorescence data analyzed using ImageJ (NIH, Rockville, MD, USA). These data confirm the significant reduction (P ≤ 0.001) of the c-Myc level caused by aPKC inhibition by ICA-1 and also ICA-1 combined with BYL.

## Discussion

The currently available chemotherapies for metastatic and advanced ccRCC often elicit a short-term response, even though the disease progression typically reverts within a year of progression-free survival (3). The currently available mTOR inhibitors (a component of the PI3K/Akt pathway) are reported to pose a limitation caused by an Akt feedback loop, which renders the therapy ineffective (14). Thus, in RCC, one could choose to target the PI3K/Akt components in combination with aPKC inhibitor (30). Alpelisib, a novel PI3Kα inhibitor, has been reported to be effective against multiple types of cancer (28). Our data suggests that the 786-0 and Caki-1 cell lines treated with a single BYL treatment caused a significant but partial inhibition of the PI3K/Akt downstream signaling pathway. In addition, BYL and aPKC inhibitor ICA-1 combination therapy is marked by a more pronounced effect on the downstream effectors (**Figure 5**).

Our investigation shows that the inhibition of PKC-ι induces the apoptosis of ccRCC. Our treatment with ICA-1 to inhibit PKC-ι and treatment with ICA-1 combined with BYL show that the pronounced effect on reducing the PKC-ι level resulted in decreased cell viability (**Figure 1**). Previous Molecular Docking studies proved that ICA-1 worked as an inhibitor of PKC-ι (31). A flow cytometry analysis revealed that a reduction of the p-PKC-ι and PKC-ι levels, as seen in the combination therapy with BYL and ICA-1, resulted in an increased level of cellular apoptosis in both the 786-0 and Caki-1 cell lines (**Figure 2**). Using a Western Blot analysis, the increased levels in cleavage of the PARP and Caspase-3 proteins indicate significant apoptosis (**Figure 3**).

Often, cancer cells with an elevated MYC expression exhibit a highly proliferative phenotype. c-Myc is overexpressed in both the Caki-1 and 786-0 cells, and it is conducive to a variety of roles such as proliferation and survival in RCC (5, 32). In the nucleus, c-Myc processes upstream oncogenic signals before carrying out the transcriptional programs that ultimately cause uncontrolled tumor development (33, 34). Although c-Myc plays a central role in different types of cancer, it is still a challenge to consider c-Myc as a direct druggable target because there is difficulty in identifying the active site as well as challenges in designing membrane permeable small molecules (10). In this study, we reported for the first time that PKC-ι is associates with c-Myc. The extent of the association is reduced as a result of combination therapy in both 786-0 and Caki-1 (**Figure 7A**). We also demonstrated that Akt1 is directly associated with PKC-ι. The extent of the Akt1 association is reduced by combination therapy in the 786-0 and Caki-1 (**Figure 7A**) cell lines. Similar trend was also observed in the association of PKC-ζ and c-Myc (**Figure 7B**).

It has been previously reported that a decreased level of c-Myc is an effect of upstream reduction of of phospho-Akt1 level (35). The decreased phosphorylation of Akt1 augments the phosphorylation of c-Myc at Thr58 by maintaining GSK3β activity. The Thr58 phosphorylation facilitates the degradation of c-Myc via proteasomal degradation (15, 36). The immunoprecipitation experiment of PKC-ι with Akt1 and c-Myc shows that there is an association of PKC-ι with Akt1 and c-Myc. These results elucidate that PKC-ι is important for activating Akt1 and c-Myc. Thus, inhibiting PKC-ι will negatively affect the activation of these proteins, which results in the amplified proteasomal degradation of c-Myc. This was evident from the enhanced effect on the c-Myc level diminution as a result of the combination drug treatment (**Figure 5**).

The Western blot analysis results with combination drug treatment and siRNA inhibition of PKC-ι suggest that there is a reduced phosphorylation of the MEK/ERK axis. Activated ERK1/2 is reported to be conducive to stabilizing the c-Myc protein via phosphorylation at Ser62 residue (15, 36, 37). We reported that in RCC, inhibiting PKC-ι with combination treatment and siRNA knockdown resulted in a considerable reduction of the phospho-Akt and phospho-ERK1/2 levels in both cell lines, thereby negatively impacting the stability of c-Myc (**Figures 6A, B**).

Wound healing and Transwell assay showed that the invasiveness and metastatic ability of the cells are distinctly impaired in combination therapy in both the 786-0 and Caki-1 cells (*P*<0.01; **Figures 9A, C**). ERK1/2 has been linked to increased invasiveness in different types of cancer (38). Increased Akt1 and ERK1/2 activation has been linked to the malignancy of renal cell carcinoma (39–41). Reduced Akt1 and ERK1/2 activation was observed in our study as a result of drug treatment and the siRNA mediated inhibition of PKC-ι. To confirm this finding, we examined the expression of the EMT markers E-cadherin, N-cadherin, phospho-vimentin and vimentin. Our results revealed that combination therapy causes a loss of N-cadherin, phospho-vimentin, and vimentin in both 786-0 and Caki-1 cells, and it promotes the E-cadherin expression in Caki-1 cells. (**Figure 9B**). Vimentin is essential for enabling mesenchymal cells to adopt rear-to-front polarity, making it a hallmark of EMT. Our previously published data confirmed the transcriptional regulation and direct association of vimentin with PKC-ι (22). PKC-ι has also been reported to promote cellular invasion and migration in several cancer types (20, 23). In this study, the pronounced inhibition of PKC-ι by ICA-1 and BYL combination therapy resulted in a decreased activation of vimentin in both cell lines. As a result, we anticipate that the inhibition of PKC-ι by either ICA-1 or by a combination of ICA-1 and BYL719, which has a more pronounced effect on PKC-ι inhibition, will lead to the reduced invasiveness and migratory ability of the cells.

**Figure 11:**
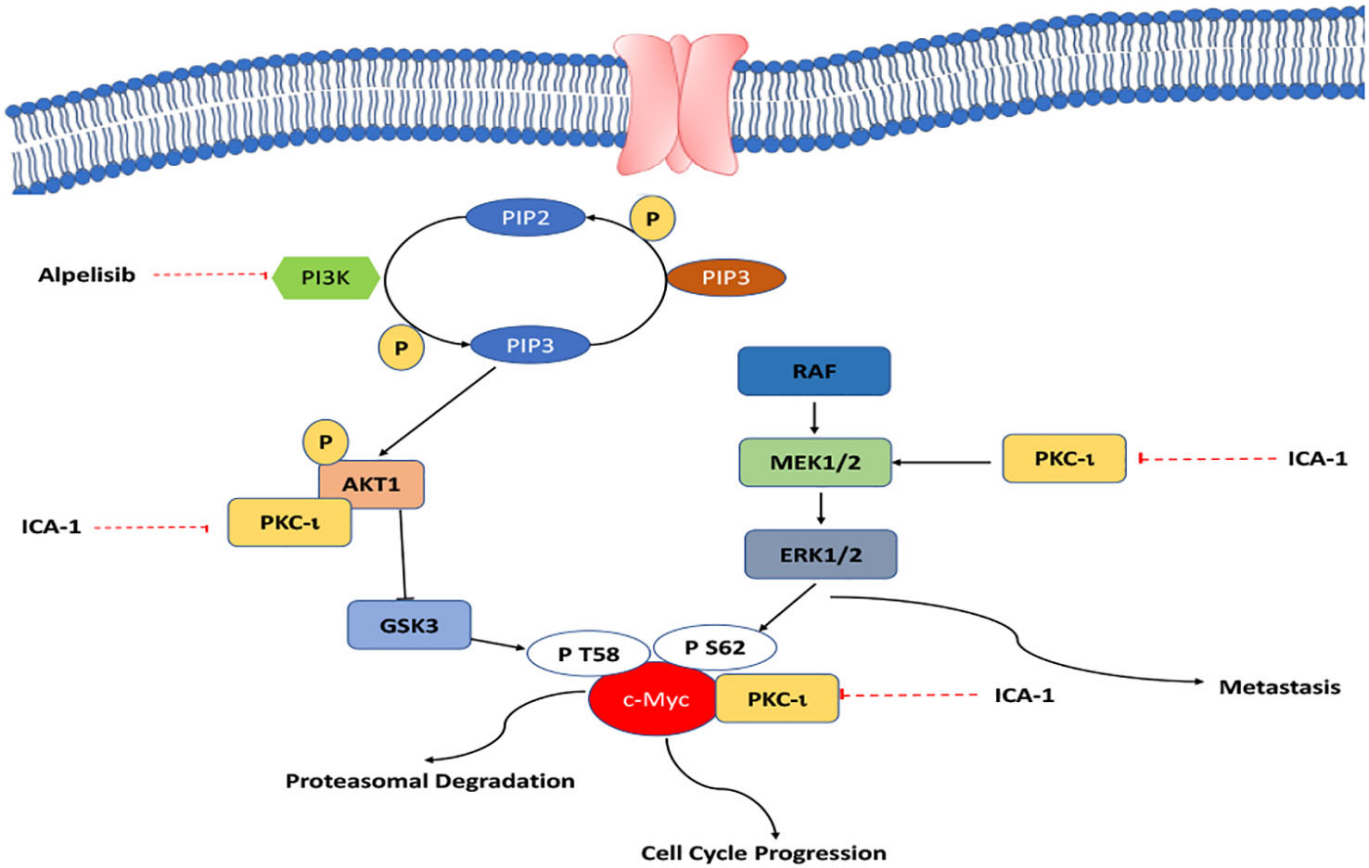
A schematic summary of how aPKCs involve the progression of ccRCC.

Our study shows that inhibiting PKC-ι can be a viable way to indirectly target c-Myc to interrupt the overactivated pathways that lead to high c-Myc expression. As PKC-ι regulates c-Myc directly and indirectly via Akt1 and ERK1/2, a combination of Alpelisib (BYL) and an aPKC inhibitor (ICA-1), is a three-pronged approach to significantly reduce PKC-ι and, thereby, c-Myc (**Figure 11**). In turn, this reduces cell proliferation, invasion, and resistance, which eventually induces apoptosis. This study provides a basis for the synergistic combination of the PI3K and aPKC inhibitors against elevated c-Myc expression. Because of the dependent nature of PKC-ι and PKC−ζ, and their association with c-Myc, PKC-ζ can also be used as a viable target to disrupt c-Myc expression in RCC using a PKC-ζ specific inhibitor, which merits further research.

## Materials and methods

### Cell lines

Human Caki-1 (ATCC^®^ HTB-46^™^) and 786-0 (ATCC^®^ CRL-1932^™^) were purchased from American Tissue Type Collection (ATCC, Rockville, MD). The Caki-1 cells are epithelial cells with adherent properties. The 786-0 cell line is a primary clear cell adenocarcinoma.

### Cell culture

Human Caki-1 and 786-O Renal cell carcinoma were cultivated in the laboratory in McCoy’s 5a Medium Modified (Catalog No. 30-2007), and RPMI-1640 Medium, ATCC 30-2001 supplemented with 10% Fetal Bovine Serum (FBS), and 1% antibiotics (Penicillin 10 U/ml and streptomycin 10 mg/ml). In turn, the cells were incubated at 37°C and 5% CO_2_. The cells were then used for the experiments a few days following subculture at 70-80% confluency.

### Compounds used

Atypical inhibitor, 1H-imidazole-4-carboxamide, 5-amino-1-[2,3-dihydroxy-4-[(phosphonooxy) methyl] cyclopentyl]-, [1R-(1a, 2b, 3b, 4a)] (ICA-1) were used for the *in-vitro* works. ICA-1 was used as an inhibitor of PKC-ι. Alpelisib (BYL719) is a selective PI3Kα inhibitor that was developed by Novartis Pharma; it was purchased from Advanced Chemblock Inc., while ICA-1 was purchased from United Chemical Resources.

### Antibodies and reagents

The antibodies to anti-Phospho PKC-ζ (T410) (2060), anti-PARP (9532), anti-Cleaved PARP (5625), anti-BCL-2 (2872), Phospho-p44/42 MAPK (Erk1/2) (Thr202/Tyr204) (9101), c-Myc (D84C12) (5605), Phospho-Akt (Ser473) (9271), Phospho-PDK1 (Ser241) (3061), PDK1 (3062) Phospho-MEK1/2 (Ser217/221) (9121), MEK1/2 (9122), Akt (pan) (40D4) (2920), Vimentin (R28)(3932), Phospho-vimentin (Ser39)(13614), N-cadherin (D4R1H) (13116) and anti-α-Tubulin (2125) were procured from Cell Signaling Technology (Danvers, MA). The antibody to Phospho PKC-ι (T555) (ab5813) was purchased from Abcam (Cambridge, MA). The Anti-Caspase-3 Antibody (sc-7272) and Anti-ERK 1/2 Antibody (C-9) (sc-514302) were purchased from Santa-Cruz (California, USA). The Cleaved Caspase 3 (Asp175) antibody (MAB835) was purchased from R&D systems (Minneapolis. MN) and the anti-PKC-ι antibody (610176) was purchased from BD Biosciences (San Jose, CA). E-cadherin (4A2C7) (33-4000) was purchased from Thermo Fisher Scientific. The immune-precipitation agarose conjugated antibodies PKC-ι (sc-376344, AKT1(sc-5398 AC) and c-Myc (sc-40AC) were purchased from Santa-Cruz (California, USA). The flow cytometry based Annexin V APC assay kits (601410) were purchased from BioVision Inc (Milpitas, CA) and Cayman Chemicals (Ann Arbor, MI), respectively. ProLong Gold Antifade Mountant with DAPI (blue) was obtained from Invitrogen Inc. (Carlsbad, CA). Enhanced Chemiluminescence (Super Signal West Pico Chemiluminescent Substrate) (34580) was purchased from Pierce (Rockford, IL). Horseradish peroxidase (HRP) conjugated goat anti-mouse (1706516), and goat anti-rabbit (1706515) secondary antibodies were bought from Bio-Rad Laboratories (Hercules, CA). The WST-1 (11644807001) reagent was purchased from Sigma-Aldrich (St. Louis, MO). The McCoys’s 5A Medium (30-2007™) was obtained from ATCC (Manassas, VA). RPMI 1640 was obtained from Corning (Manassas, VA). ProLong Gold Antifade Mountant with 4′,6-diamidino-2-phenylindole (DAPI) (blue) and Alexa Fluor 568 (red) conjugated donkey anti-rabbit secondary antibody were purchased from Invitrogen Inc. (Carlsbad, CA, USA).

### WST-1 assay for cell viability and cytotoxicity

To assess the vitality and proliferation of the cell cultures, the WST test was utilized (42). 96-well plates with around 2000 cells in each well were used to plate the 786-0 and Caki-1 cell lines. Fresh media were provided (200µL/well) 24 hours after the plating occurred. The cells were exposed to ICA-1 in varying micro-molar, BYL, and ICA-1 combined with BYL concentrations for three days. After three days of drug treatment, the cell viability was assessed. At the end of the treatment, the old media were discarded, and the fresh media (180µl) and WST-1 reagent (20µl) were added to each well. After three hours of incubation, we measured the absorbance at 480 nm on the plate while the reference background wavelength was set at 630 nm. The average of the absorbance for triplicate readings for each sample was taken, and the culture medium background was subtracted from the assay to obtain the corrected absorbance. The corrected absorbance is assumed to be proportional to the cell viability. Assuming that the control has a 100% cell viability, a dose-response curve for ICA-1 was generated using both cell lines.

The following formula is used to compute the coefficient of drug interaction (CDI):

CDI = AB/(A×B). The ratio of the combination groups to the control group is given by the absorbance of each group, and the ratio of the single agent group to the control group is given by A or B. Accordingly, a CDI value of 1, = 1, or >1 denotes a drug’s additive, antagonistic, or synergistic effects (43).

### Annexin-V-FITC and PI analysis

The Annexin-V/APC and DAPI-based flow cytometry was used to distinguish the apoptotic population from the healthy population. For both cell lines, approximately 5x10^4^ cells were cultured in 100 mm flasks. Subsequently, 24 h post-plating, fresh media were supplied, and the cells were treated with ICA-1, BYL, or the combination of BYL with ICA-1. Additional doses were supplied every 24 h across the 3-day incubation period. Next, the cells were lifted from 100mm round flasks using a Hyclone HyQtase cell lifting solution. Subsequently, media was added to the cells and the cells were centrifuged. Subsequently, the supernatant was removed, and the cell pellets were collected. The cells were stained according to the manufacturer’s instructions with Annexin V- fluorescein isothiocyanate (FITC), and they were co-stained with propidium iodide (PI) (Invitrogen). Subsequently, the stained cells were immediately analyzed via Canto II (BD Biosciences) and FACsDIVA 6.3.1 software (50,000 events were collected per sample).

### Cell lysate preparation and immunoblot analysis

Both the Caki-1 and 786-0, 5x10^4^ cells were plated in 150mm plates and dosed with the Annexin V assay as described in section 2.6. After the treatment was completed, the plates containing the cells were placed in ice and the media was discarded. Afterward, the cells were washed with Dulbecco’s Phosphate Buffered Saline (DPBS), scraped in a 1.5µL Eppendorf tube and suspended in 500µL of a cell lysis buffer. Subsequently, the concentration of the protein samples was measured using the Bradford assay. Approximately 40-80 µg of proteins were loaded in each well of the Sodium dodecyl sulfate (SDS) Page gel. Finally, electrophoresis was carried out according to the method described in the previously published protocol (23). Primary antibodies were used to determine the expression of phospho-PKC-ι (1:1000), PKC-ι (1:1000), phospho−PKC-ζ (1:1000), PKC-ζ (1:500), p-Erk1/2 (1:1000), Anti-ERK ½ (1:1000), c-Myc (1:1000), Phospho-Akt (Ser473) (1:1000), p-MEK1/2 (Ser.217/221) (1:1000), MEK1/2 (1:1000), Akt (1:1000), N-cadherine (1:1000), E-cadherine (1:1000), p-Vimentin (Ser39) (1:1000), Vimentin, PARP, cleaved PARP, Caspase-3 (1:1000), cleaved Caspase-3 (1:1000), Bcl-2 (1:1000) and Bcl-xl (1:1000).

### Wound healing assay

This experiment was performed as per our previously published protocol (21, 23). The drug treatment was performed as described in Section 4.6.

### Trans-well invasion and migration assay

The cells were serum starved in a T75 flask for 24h Next, the cells were detached from the flask’s surface by using trypsin for two minutes, and they were re-suspended in a 10% serum-containing media before being plated (5 X 10^4^) into the upper chamber of the 24 wells Transwell permeable support (pore size: 8μm) that had been coated with 250µg/ml of Basement Membrane Extract (BME) solution. The serum-containing media (with 20%FBS) was loaded into the receiver plate (lower chamber) as a chemoattractant. The 786-0 and Caki-1 cells in the upper chamber were treated with DMSO control, ICA-1, BYL, or BYL combined with ICA-1 for three consecutive days. Following treatment, the invasive cells of the lower chamber were stained with crystal violet and the photographs were taken with 1X resolution using a microscope. These photographs were taken where there was colony formation or where maximum cell density was observed. The number of the cells in the field was quantified using Image J.

### Transfection of Caki-1 and 786-0 cells and RNA interference

Approximately 1×10^5^ cells were inoculated into 100mm of tissue and placed on a culture plate. Twenty-four hours post-plating, the cells were transfected using 40nM of PKC-ι, and siRNA was used to knock down the *PRKCI* gene. Following 8 h of incubation in a serum-free media, the cells were supplied with a 10% serum-containing medium. Subsequently, after 48 h of incubation, the cell lysate was collected and subjected to an immunoblot analysis to determine the expressions of PKC-ι, PKC-ζ, p-Erk1/2, Anti-ERK 1/2 Antibody, c-Myc, Phospho-Akt (Ser473), p-PDK1 (Ser241), p-MEK1/2 (Ser.217/221), MEK1/2, and Akt.

### Immunoprecipitation

PKC-ι and PKC-ζ were immunoprecipitated (IP) from 1000μg of proteins collected from cell lysate suspensions. An agarose conjugated PKC-ι and PKC-ζ primary antibody was used, and the manufacturer’s protocol was followed to immunoprecipitate the protein. The precipitated proteins were separated by SDSPAGE and, finally, they were analyzed by using the Western blot technique to determine the associated proteins that had the PKC-ι and PKC-ζ proteins.

### c-Myc and 4′,6-diamidino-2-phenylindole staining

The cells were grown in 4 well chamber slides. Twenty-four hours after plating, the 786-0 and Caki-1 cells were treated with ICA-1, BYL, or BYL combined with ICA-1 for three consecutive days. Post-treatment, the media was removed, and the cells were washed with DPBS and fixed with 4% paraformaldehyde for 15 minutes. Subsequently, the paraformaldehyde was removed by washing it three times with DPBS. Then, the slides were blocked with 10% FBS in PBS for 30 minutes. The primary c-Myc antibody was made in 1.5% Bovine Serum Albumin (BSA) in DPBS (1:50 dilution), and the slides were incubated overnight. After removing the primary antibody, the slides were washed three times with DPBS and incubated with the Alexa Fluor 647 (Red) (1:200) for thirty minutes. Next, DAPI was used to stain the nuclei for 30 min. Next, the nuclei were stained with DAPI for 30 min. The coverslips were mounted on glass slides, and they were inspected under a Zeiss LSM 510 Meta confocal microscope with a magnification of 20x.

### Quantitative real-time PCR

RNA was isolated from the siRNA treated 786-0 and Caki-1 cell lysates, as described in Section 4.11. qPCR was performed, as previously published (22). The following primers were used in the experiment: PKC-ζ; forward: ACCCCTTCCTGGTCGGATTA; reverse: AGGGGGCTTCTGGAAGAGTA. PKC-ι; forward: TTGCAATGAGGTTCGAGACA; reverse: CTGAGATGATACTGTACACGGG. C-Myc; forward ACACCCTGCAATCTTTCAGACA; reverse GATTCCACTTTGCGTTCAAGGT. β-actin; forward: AGAGCTACGAGCTGCCTGAC and reverse; AGCACTGTGTTGGCGTACAG was used as the housekeeping gene.

### Statistical analysis

All the data are presented as mean ± SEM. Statistical analysis was performed with one or two-way ANOVA followed by a Tukey’s HSD test as the multiple comparisons tests using the GraphPad Prism software for statistical analysis. A *P* value of less than or equal to 0.05 indicated statistical significance.

## Data availability statement

The datasets presented in this study can be found in online repositories. The names of the repository/repositories and accession number(s) can be found in the article/**Supplementary Material**.

## Ethics statement

Ethical approval was not required for the studies on humans in accordance with the local legislation and institutional requirements because only commercially available established cell lines were used. Ethical approval was not required for the studies on animals in accordance with the local legislation and institutional requirements because only commercially available established cell lines were used.

## Author contributions

KK and MA-D: conceptualization; KK and WR: formal analysis; KK and MA-D: investigation; KK, CA, and MA-D: methodology; KK: writing-original draft; KK, WR, and MA-D: writing-review and editing; MA-D resources; MA-D supervision; MA-D funding acquisition. All authors contributed to the article and approved the submitted version.
